# Spatiotemporal heterogeneity and long-term impact of meteorological, environmental, and socio-economic factors on scrub typhus in China from 2006 to 2018

**DOI:** 10.1186/s12889-023-17233-y

**Published:** 2024-02-21

**Authors:** Jiaojiao Qian, Yifan Wu, Changqiang Zhu, Qiong Chen, Hongliang Chu, Licheng Liu, Chongcai Wang, Yizhe Luo, Na Yue, Wenhao Li, Xiaohong Yang, Jing Yi, Fuqiang Ye, Ji He, Yong Qi, Fei Lu, Chunhui Wang, Weilong Tan

**Affiliations:** 1 Department of Epidemiology, School of Public Health, Nanjing Medical University, Nanjing, China; 2 Nanjing Bioengineering (Gene) Technology Center for Medicines, Nanjing, China; 3 Center for Disease Prevention and Control of Jiangsu Province, Nanjing, Jiangsu China; 4 Hainan International Travel Healthcare Center, Haikou, Hainan China; 5 Department of Transfusion Medicine, Xijing Hospital, Fourth Military Medical University, Xi’an, Shaanxi China; 6 Xiamen International Travel Health Care Center (Xiamen Customs Port Outpatient Department), Xiamen, China; 7 College of Information Engineering, Zhejiang University of Technology, Liuhe Rd. 288, Hangzhou, 310023 China

**Keywords:** Scrub typhus, Bayesian model, Geodetector, Spatiotemporal heterogeneity, Meteorological and socio-economic factors, Environment

## Abstract

**Background:**

Large-scale outbreaks of scrub typhus combined with its emergence in new areas as a vector-borne rickettsiosis highlight the ongoing neglect of this disease. This study aims to explore the long-term changes and regional leading factors of scrub typhus in China, with the goal of providing valuable insights for disease prevention and control.

**Methods:**

This study utilized a Bayesian space–time hierarchical model (BSTHM) to examine the spatiotemporal heterogeneity of scrub typhus and analyze the relationship between environmental factors and scrub typhus in southern and northern China from 2006 to 2018. Additionally, a GeoDetector model was employed to assess the predominant influences of geographical and socioeconomic factors in both regions.

**Results:**

Scrub typhus exhibits a seasonal pattern, typically occurring during the summer and autumn months (June to November), with a peak in October. Geographically, the high-risk regions, or hot spots, are concentrated in the south, while the low-risk regions, or cold spots, are located in the north. Moreover, the distribution of scrub typhus is influenced by environment and socio-economic factors. In the north and south, the dominant factors are the monthly normalized vegetation index (NDVI) and temperature. An increase in NDVI per interquartile range (IQR) leads to a 7.580% decrease in scrub typhus risk in northern China, and a 19.180% increase in the southern. Similarly, of 1 IQR increase in temperature reduces the risk of scrub typhus by 10.720% in the north but increases it by 15.800% in the south. In terms of geographical and socio-economic factors, illiteracy rate and altitude are the key determinants in the respective areas, with *q*-values of 0.844 and 0.882.

**Conclusions:**

These results indicated that appropriate climate, environment, and social conditions would increase the risk of scrub typhus. This study provided helpful suggestions and a basis for reasonably allocating resources and controlling the occurrence of scrub typhus.

**Supplementary Information:**

The online version contains supplementary material available at 10.1186/s12889-023-17233-y.

## Background

Scrub typhus is a natural focus disease caused by *Orientia tsutsugamushi*, an obligate intracellular bacterium transmitted by bites of infected larval mites. This disease is characterized by fever, eschar, rash, and lymph node enlargement, and can even lead to multiple organ failure and death in severe cases with a fatality rate of up to 70% [1]. *O.tsutsugamushi* is geographically endemic across vast areas of Asia and islands in the Pacific and Indian Oceans. Scrub typhus is one of the most widespread and severe infections of *rickettsia,* resulting in over one million infective cases each year [1, 2]. Recent research [3–7] has shown that the distribution of scrub typhus is no longer limited to the Asia–Pacific region, but has also been documented in other regions, such as Dubai, Kenya, and South America. Due to the lack of an effective vaccine and rapid diagnosis, scrub typhus continues to pose a serious public health threat.

In China, scrub typhus was initially confined to tropical and subtropical areas of southern China after its identification in Guangzhou province in 1948 [8]. However, the first case of autumn–winter scrub typhus occurred in Mengyin County, Shandong Province, in 1986, indicating its spread to northern China [9]. Since then, new natural foci have been continuously reported and identified, leading to a significant increase in geographical distribution and a noticeable upward trend in the number of cases annually. Several studies have shown that there was initially spatial and temporal heterogeneity of scrub typhus between southern and northern China [10, 11].

Previous studies have found significant correlations between climate factors and the spatiotemporal dynamics of scrub typhus [10, 12]. For instance, scrub typhus in southern China and northern Japan primarily occurs during the summer [13, 14], while in northern China and South Korea, it mainly occurs in autumn and winter [13, 15]. Several studies [16–20] have investigated the relationship between climate factors and the epidemiology of scrub typhus using statistical models. Ding et al. [12] examined the impact of climate on the spatiotemporal dynamics of scrub typhus through a generalized additive mixed model. Zheng et al. [20], analyzed the spatial heterogeneity of scrub typhus in southern China using an enhanced regression tree modeling procedure that considered multiple spatial correlations. They concluded that the spread of scrub typhus was highly influenced by temperature and humidity. Wei et al. [21] employed a time series Poisson regression model and a distributed hysteresis nonlinear model to establish the association between precipitation, relative humidity and scrub typhus in Guangzhou.

This article addresses the lack of research on the overall spatiotemporal dynamics of scrub typhus in China, particularly the impact of environmental and socio-economic factors. The study utilizes the Bayesian spatiotemporal stratification model and the Geodetector model to analyze the long-term perspective (2006–2018) of scrub typhus risk in southern and northern China. Additionally, the study quantifies the influence of meteorological, environmental, and socio-economic factors on scrub typhus. The finding also identifies provincial hot and cold spots, offering valuable insights into the health threat caused by scrub typhus.

## Methods

### Ethics statement

This study was approved by the ethics committee of Nanjing Bioengineering (Gene) Technology Center for Medicines (No:2021BY07). Patient consent was not required because no patients’ individual information was included in this study and population data were collected from the public database of China.

### Study region

**Fig. 1:**
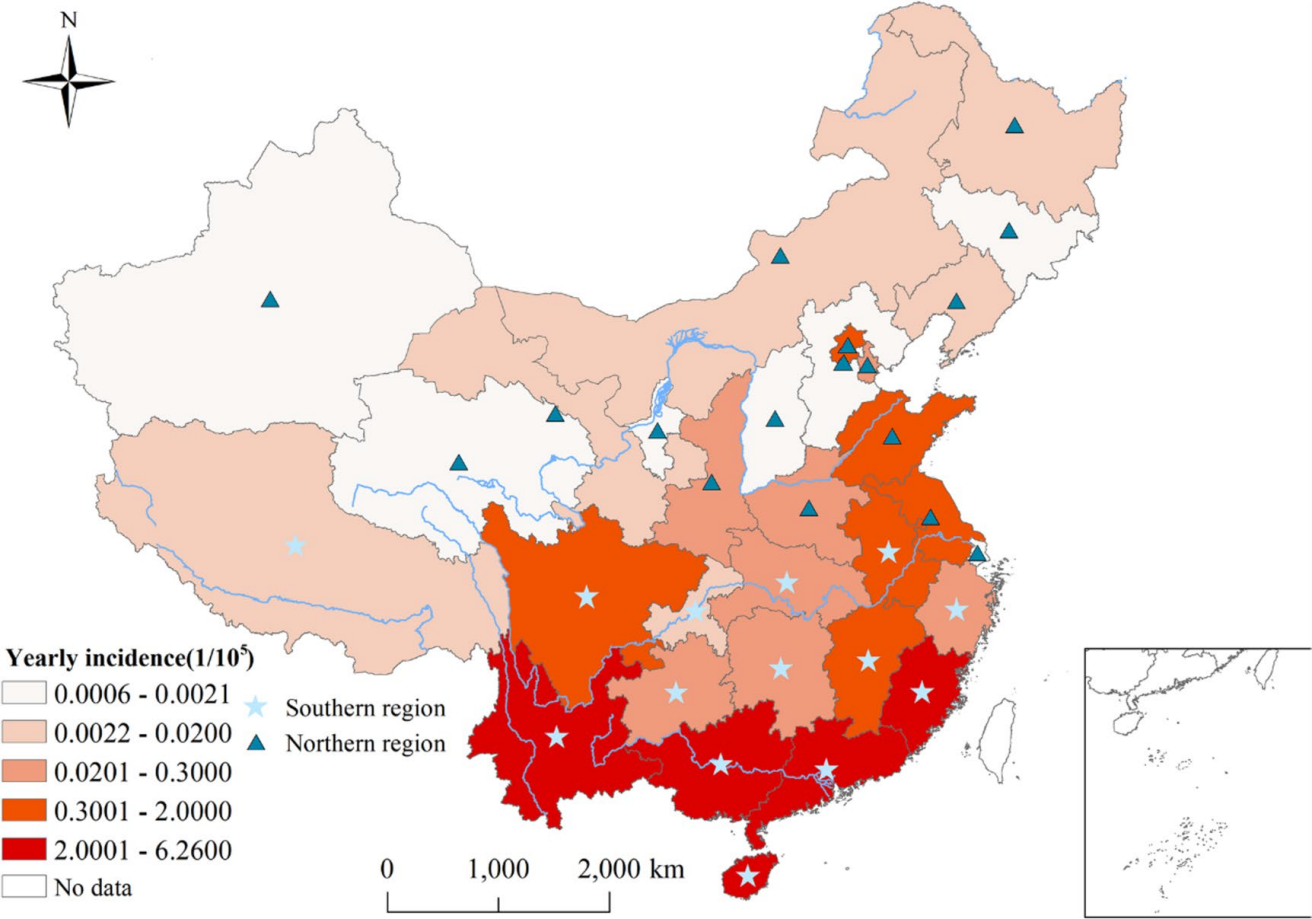
Yearly incidence of scrub typhus between 2006 and 2018 in China

In China, *Leptotrombidium* *delicense* (*L.* *delicense)* and *Leptotrombidium scutellare* (*L. scutellare)* are the two most important species of mites [22, 23]. L. scutellare is widely distributed in China and is the dominant mite species in the north of the Yangtze River. L.delicense is the main vector of summer scrub typhus in southern China, mainly distributed in provinces south of 30° north latitude in China [24, 25]. Therefore, in our study, 31 provinces in China are divided into two research areas, the provinces south of 30° N (including those passing through 30° N) are the south, and the north of 30° N are the north (Fig. 1): fourteen provinces in southern (Tibet, Anhui, Hubei, Zhejiang, Jiangxi, Hunan, Yunnan, Guizhou, Fujian, Guangxi, Guangdong, Hainan, Chongqing, and Sichuan) and seventeen provinces in northern (Jiangsu, Shanghai, Beijing, Hebei, Ningxia, Henan, Shandong, Inner Mongolia, Shanxi, Shaanxi, Gansu, Qinghai, Tianjin, Liaoning, Jilin, Xinjiang, and Heilongjiang).

### Data collection

**Fig. 2:**
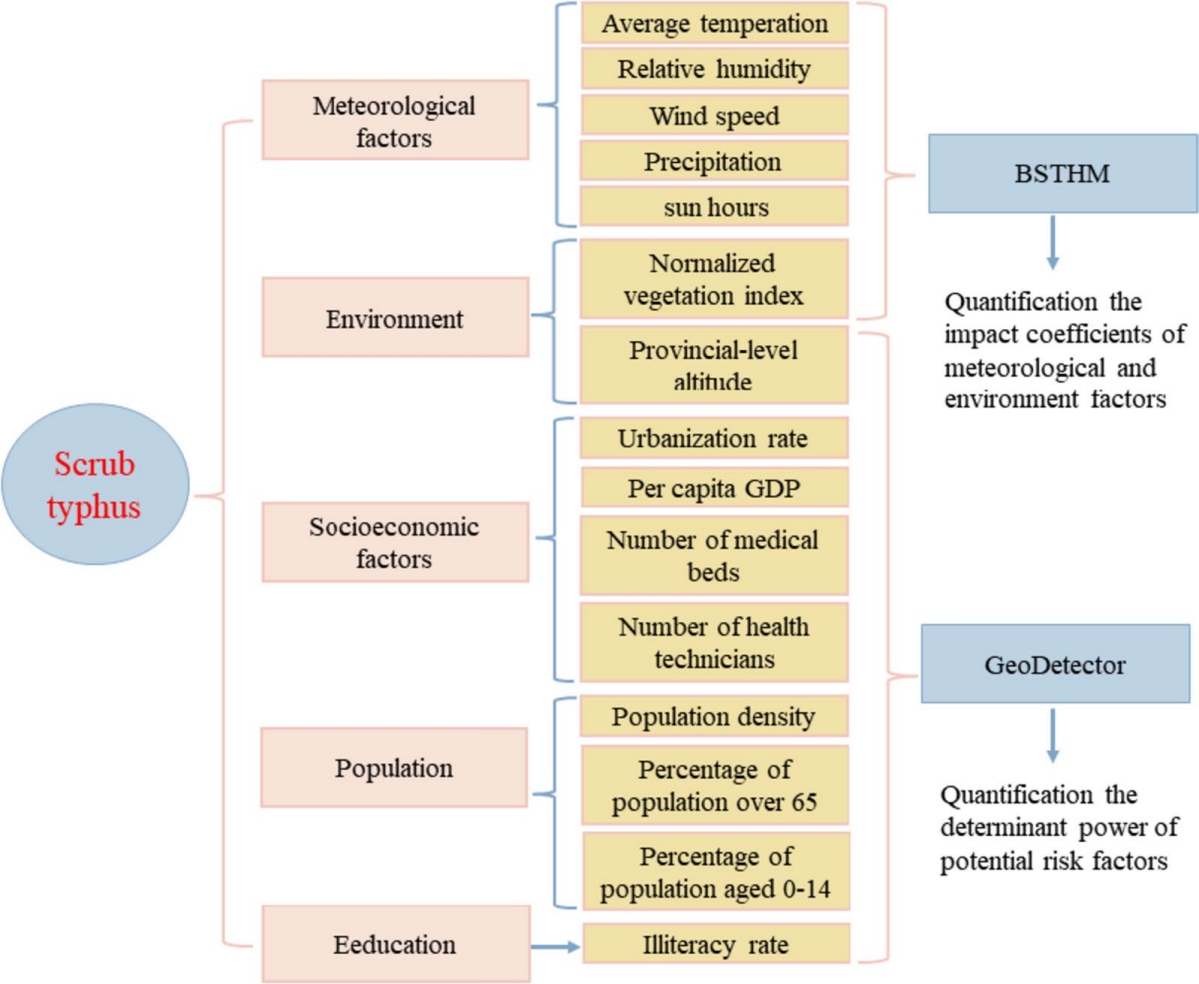
Potential drivers of scrub typhus and the statistical models used in this study

Monthly data on scrub typhus cases for the period from January 2006 to December 2018 were obtained from the Chinese Center for Disease Control and Prevention (https://www.phsciencedata.cn/Share/). Monthly meteorological data, namely average temperature, precipitation, relative humidity, wind speed, and hours of sunlight, for the same period were collected from the China Meteorological Data Sharing Service System (https://data.cma.cn/data/) (Fig. 2). The average data of vegetation normalization index (NDVI) were collected from the MOD13A3 (10.5067/MODIS/MOD13A3.006). The average data of altitude was collected from the Geospatial data cloud (http://www.gscloud.cn/). Yearly socioeconomic variables data, including per capita gross domestic product (GDP) (10^4^CNY), urbanization rate, population density, number of health technicians per 1000 persons, illiteracy rate, number of medical beds per 1000 persons, percentage of population aged 0–14, and percentage of population over 65 were acquired from the Chinese economic Statistical Yearbook (http://www.stats.gov.cn/) (Fig. 2). Specific data can be found in Supplementary Document S1. The case definition was based on the unified diagnostic criteria formulated by the Chinese Ministry of Health (MOH). See supplementary material S2 for detailed diagnostic criteria.

### Bayesian space–time hierarchy model (BSTHM)

The spatiotemporal phenomenon revealed the changing trend of scrub typhus risk in time and space, which was decomposed into two parts: global and local components [26]. The global part represented a common spatiotemporal variation of scrub typhus, whereas the local part revealed the spatiotemporal heterogeneity of the incidence of scrub typhus throughout the whole study period. Specifically, we used the model [26, 27] with the Poisson distribution to capture spatial hotspots and coldspots of scrub typhus and the quantity association between the incidence of this disease and the environmental factors (Fig. 2). In the model, we let ${y}_{it}$,${n}_{it}$, and ${u}_{it}$ represent the new scrub typhus cases in province $i(=\mathrm{1,2},\dots ,31)$ at time point $t(=\mathrm{1,2},\dots ,156)$, the total population at the end of a year, and the relative risk of scrub typhus incidence, as follows:

$${y}_{it}\sim Poisson({n}_{it}{u}_{it})$$

$$\mathrm{log}\left({u}_{it}\right)=\alpha +{s}_{i}+{b}_{0}{t}^{*}+{v}_{t}+{b}_{1i}{t}^{*}+{\varepsilon }_{it}$$

where α is the overall log risk of scrub typhus in China and ${t}^{*}=t-78$ (centering at the mid-observation period). The $\mathrm{exp}({s}_{i})$ is the spatial risk of this disease, which is influenced by some related factors in the study period, such as economic conditions, local prevention and control policies, and medical resources. ${(b}_{0}{t}^{*}+{v}_{t})$ describes the overall time trend common to all provinces with ${v}_{t}\sim N(0,{\sigma }_{v}^{2})$, which allows for the nonlinearity of the overall trend pattern. The term ${b}_{1i}{t}^{*}$ allows each province to have its own trend. It can be used to capture the deviation from ${b}_{0}$ for each region. For example, if ${b}_{1i}>0$, then the province $i$ has a stronger temporal trend than the general trend of the total region. The last term ${\varepsilon }_{it}\sim N(0,{\sigma }_{\varepsilon }^{2})$ [28] is the Gaussian random noise variable and captures additional variability not yet explained by other model components. The prior distribution of the global spatial random effect term ${s}_{i}$ is the BYM model [29]. A strictly positive half Gaussian prior ${N}_{+\infty }(\mathrm{0,10})$ was given to all random effect standard deviations. In this study, we used the conditional autoregressive (CAR) prior with a space adjacency matrix ${W}_{31\times 31}$ to impose spatial structure, if the country $i$ and $j$ shared a common border, then ${W}_{ij}=1$, otherwise, ${W}_{ij}=0$. CAR before spatial random effect showed that the overall risk of disease in neighboring provinces was similar. ${b}_{1i}{t}^{*}$ has the same BYM prior as ${s}_{i}$. In Bayesian simulation, any interval containing 95% of a posteriori quality is a frequency confidence interval (CI), usually called a credible interval (CRI), and sometimes also called a Bayesian confidence interval. In general, the 2.5th and 97.5th percentiles of the posterior sample were chosen as the 95% CRI.

Based on the posterior parameters of the BSTHM, we classify countries into nine categories(3 risk categories × 3 trend categories) according to a two-stage classification rule [27]. In the first stage, a province is defined as a hotspot if the posterior probability $P(exp({s}_{i})>1|data)\ge 0.8$; a province is defined as a coldspot if $P(exp({s}_{i})>1|data)\le 0.2$; if $0.2<P(exp({s}_{i})>1|data)<0.8$, the province is defined as neither hotspots nor coldspots. In the second stage, according to the local slopes ${b}_{1i}$, we further classify each risk category in the first stage into three trend patterns: level 1, the increase in the risk of scrub typhus is faster than the mean trend if $P({b}_{1i}>0|{h}_{i,}data)\ge 0.8$; level 2, the increasing in the risk of this disease is slower than the overall trend if $P({b}_{1i}>0|{h}_{i,}data)\le 0.2$; level 3, the increasing in the disease has no difference with the mean level if $0.2<P({b}_{1i}>0|{h}_{i,}data)<0.8$. The whole BSTHM was performed in OpenBUGS [27]. We ran two Markov chain Monte Carlo (MCMC) chains for 20,000 iterations and discarded the first 5000 iterations as aging. The diagnosis of convergence of Bayesian estimation was evaluated by Brooks Gelman Rubin (BGR) ratio [30]. The closer the ratio is to 1.0, the better the model converges [26]. Of the total 273 parameters of the Bayesian spatiotemporal model, only 0.73% had a BGR ratio greater than 1.05.

### GeoDetector q statistics

GeoDetector is a spatial variance analysis method using the q-statistic, which can be used to quantify the powers between scrub typhus and potential risk factors [31–33] (Fig. 2). It was expressed as:

$$q=1-\frac{q}{N{\sigma }^{2}}\sum_{h=1}^{L}Nh{\sigma }_{h}^{2}$$

Where $q$ represents the non-linear relation between the decisive socioeconomic factors and scrub typhus. The value ranges from 0 to 1, and a higher value of q-statistic suggests a higher determinant power of a risk factor or the heterogeneity of a target variable. $N$ and ${N}_{h}$ are the numbers of provinces in the total study area and the $h$-th stratum ($h=\mathrm{1,2},\dots ,L)$, respectively.${\sigma }^{2}$ and ${\sigma }_{h}^{2}$ represent the variances in scrub typhus incidence in the entire countries in the $h$-th stratum, respectively.

The issue of modifiable area unit problem (MAUP) is crucial for exploring spatiotemporal epidemiology. We optimize the parameters based on the optimal parameters-based geographical detector (OPGD) model developed by Song et al. [34] in 2020. The parameters optimization consists of the optimization of spatial discretization and optimization of spatial scale. This study selects the optimal combination of discretization methods and the break number for each geographical continuous variable as the optimal discretization parameter. Q values of variables are major contributors to the scrub typhus incidence, and they reach the maximum values when the spatial unit is 100 km. The 90% quantiles of Q values show a similar trend. Therefore, we chose 100 km as the optimal spatial unit for spatial analysis. On this basis, select the optimal parameter combination for spatial discretization for each continuous variable. The specific discretization method selection and interrupt number are shown in the supplementary file S3. Analyses were completed using the “GD” package in R4.3.1.

## Results

### Descriptive analysis

**Fig. 3:**
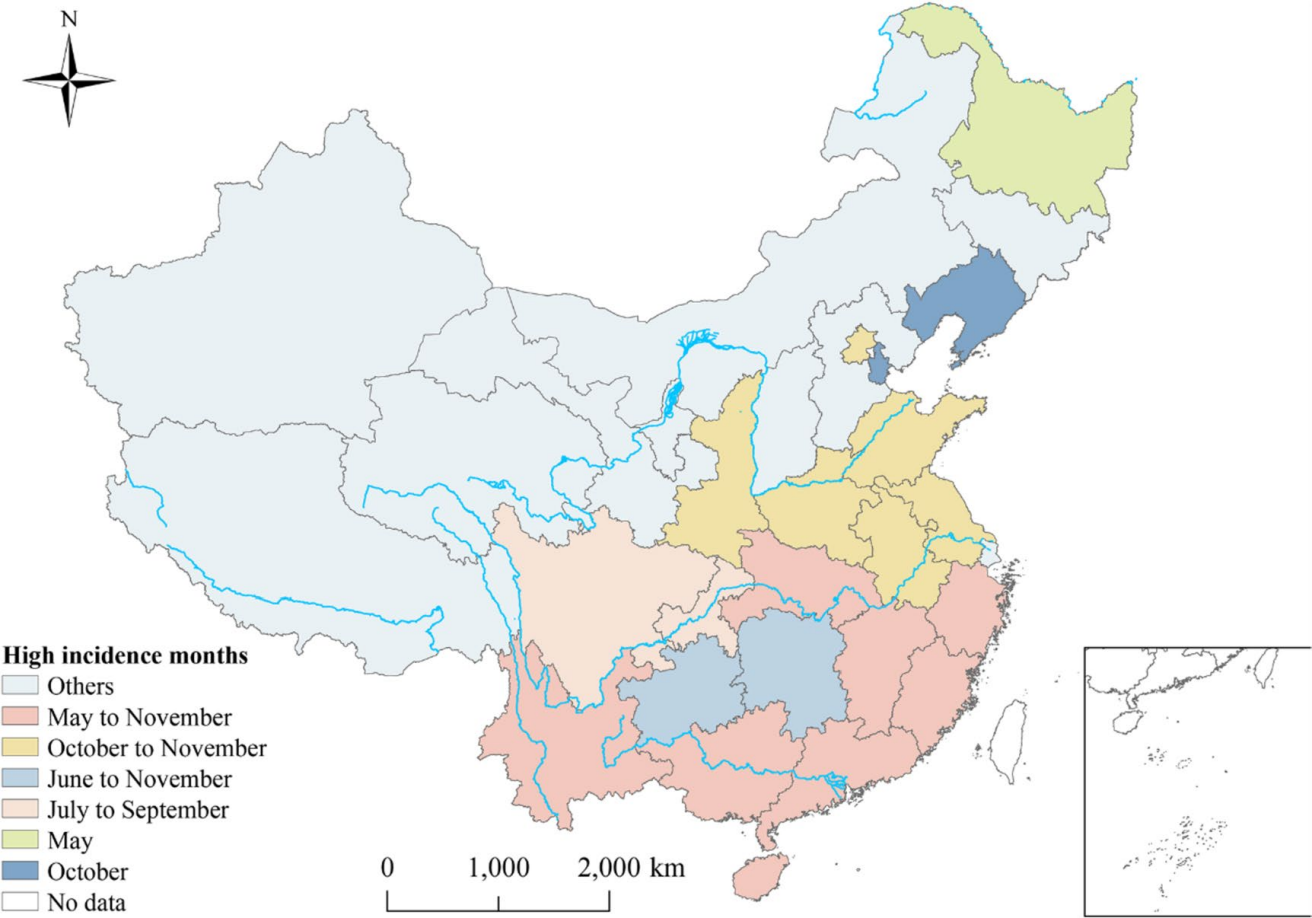
Temporal distribution of scrub typhus in 31 provinces

Between January 2006 and December 2018, a total of 142,849 cases of scrub typhus were reported in the study regions, over a span of 156 months. The monthly incidence rate was 0.067 per 100,000 people. Prior to 2012, the incidence rate was slightly higher among men compared to women. However, after 2012, the incidence among women surpassed that of men significantly (Table 1). Among different age groups, the highest incidence rates were observed in children under 10 years old and people over 50 years old. The majority of scrub typhus cases were reported among farmers, scattered children, individuals engaged in housework and unemployment, and students (Table 1). Figure 3 showed the unique seasonality of scrub typhus in each province. For instance, in Hunan and Guangdong, the highest incidence season occurred during summer and autumn, from June to November (Fig. 3). Please refer to the supplementary figure in Annex S4 for the trend in monthly incidence rate and the number of cases.

**Table 1:** Characteristics of scrub typhus cases in China from 2006 to 2018

Group	2006	2007	2008	2009	2010	2011	2012	2013	2014	2015	2016	2017	2018
Sex (1/10^5^)													
Male	3.29	3.45	6.46	7.83	9.76	14.11	19.00	21.64	30.05	32.58	39.81	40.56	46.98
Female	2.86	2.96	5.62	6.99	8.67	13.54	20.00	26.33	36.13	36.59	45.71	46.51	53.40
Age (1/10^5^)													
0-	2.31	2.14	3.74	5.82	6.75	8.55	11.86	13.57	16.09	14.14	18.90	20.03	24.06
10-	0.16	0.18	0.26	0.49	0.50	0.66	0.89	0.95	1.56	1.33	1.68	1.77	2.32
20-	0.15	0.19	0.40	0.45	0.52	0.74	0.83	1.09	1.51	1.44	1.97	1.93	2.25
30-	0.26	0.30	0.49	0.73	0.93	1.04	1.59	1.90	2.87	2.89	3.69	4.03	4.95
40-	0.42	0.42	0.99	1.18	1.39	2.02	2.76	3.40	4.46	4.86	5.79	5.95	7.00
50-	0.69	0.69	1.32	1.46	1.91	2.82	4.86	6.03	8.82	9.63	12.40	12.96	15.45
60-	0.82	0.92	1.67	1.73	2.58	4.10	6.20	8.28	11.91	14.44	16.38	17.39	19.95
70-	0.73	0.89	1.78	1.67	2.10	3.95	6.02	7.29	11.25	12.57	14.34	13.58	14.56
80-	0.61	0.69	1.42	1.30	1.75	3.76	3.99	5.47	7.70	7.88	10.37	9.43	9.83
Case fatality rate(1/10^5^)	0	75.08	115.74	0	269.28	99.67	100.89	36.02	49.90	17.36	55.32	26.60	22.42
Occupation(case)													
Farmers	728	855	1687	2078	2719	4249	6173	7847	11840	13224	16772	17546	-
Scattered children	90	98	198	312	394	478	705	788	939	839	1078	1123	-
Housework and unemployment	45	38	98	117	122	204	441	639	922	888	1081	1111	-
Students	136	120	162	300	289	320	448	521	678	563	785	849	-
Retiree	46	34	67	45	81	125	197	275	326	427	449	431	-
Works	55	48	117	101	114	125	176	247	317	305	368	359	-

### Environmental characteristics

The average values of rainfall, temperature, humidity, and NDVI in the southern region were 112.74-mm, 17.33 ℃, 73.85%, and 58.94%, respectively, which were higher than the corresponding values in the northern region (Tables 2 and 3). Conversely, the wind speed and sunshine hours in the northern region were higher than those in the southern region, with average values of 2.34-m/s and 197.91-h, respectively (Tables 2 and 3).

**Table 2:** Descriptive statistics for environmental variables in northern China from January 2006 to December 2018

Environmental variables	Mean	Min	Max	Percentiles		
				25	50	75
Monthly precipitation (mm)	48.72	0.00	486.40	7.90	27.69	70.56
Monthly average temperature (℃)	9.97	-23.21	31.96	1.00	11.43	20.52
Monthly relative humidity (%)	60.09	27.43	86.30	51.03	60.60	69.37
Monthly wind speed(m/s)	2.34	1.06	4.04	2.00	2.30	2.63
Monthly total sun hours (h)	197.91	32.90	325.45	166.39	198.59	231.15
Monthly normalized vegetation index (%)	36.11	2.90	83.90	20.00	31.85	50.03

**Table 3:** Descriptive statistics for environmental variables in southern China from January 2006 to December 2018

Environmental variables	Mean	Min	Max	Percentiles		
				25	50	75
Monthly precipitation (mm)	112.74	0.14	982.00	38.63	92.94	160.03
Monthly average temperature (℃)	17.33	-6.56	30.69	11.26	18.31	24.31
Monthly wind speed (m/s)	1.96	0.80	3.77	1.64	1.90	2.20
Monthly relative humidity (%)	73.85	27.67	89.33	70.63	76.45	80.40
Monthly total sun hours (h)	146.46	11.43	303.97	107.79	145.02	187.03
Monthly normalized vegetation index (%)	58.94	13.00	80.20	50.80	62.80	71.40

### Temporal heterogeneity

**Fig. 4:**
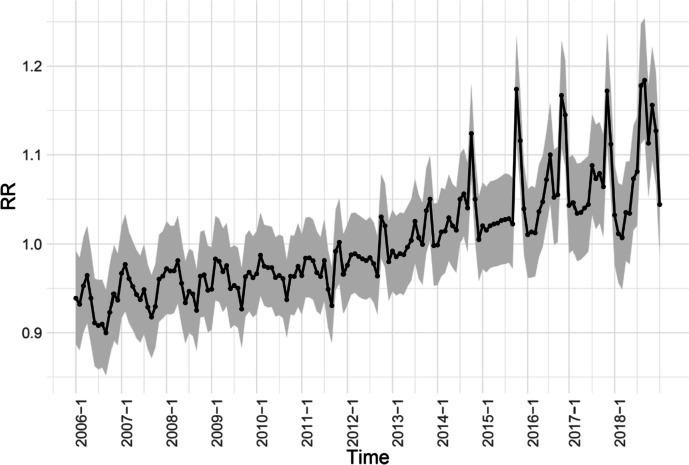
Monthly temporal relative risks of scrub typhus from 2006 to 2018. ${(b}_{0}{t}^{*}+{v}_{t})$ describes the overall time trend common to all provinces with ${v}_{t}\sim N(0,{\sigma }_{v}^{2})$

Temporally, the overall risk of scrub typhus was calculated by BSTHM for 31 provinces in terms of time dimension. The analysis revealed an upward trend and significant seasonality (Fig. 4). The incidence of scrub typhus showed a significant increase in autumn (September to November), with an average relative risk (RR) of 1.021. The highest risk was observed in October, with an average RR of 1.046. Additionally, a secondary peak was observed in summer (June to August), with an average RR of 1.002. In contrast, the risk was significantly lower in winter and spring, with an average RR of 0.992 (Fig. 4).

### Spatial heterogeneity

**Fig. 5:**
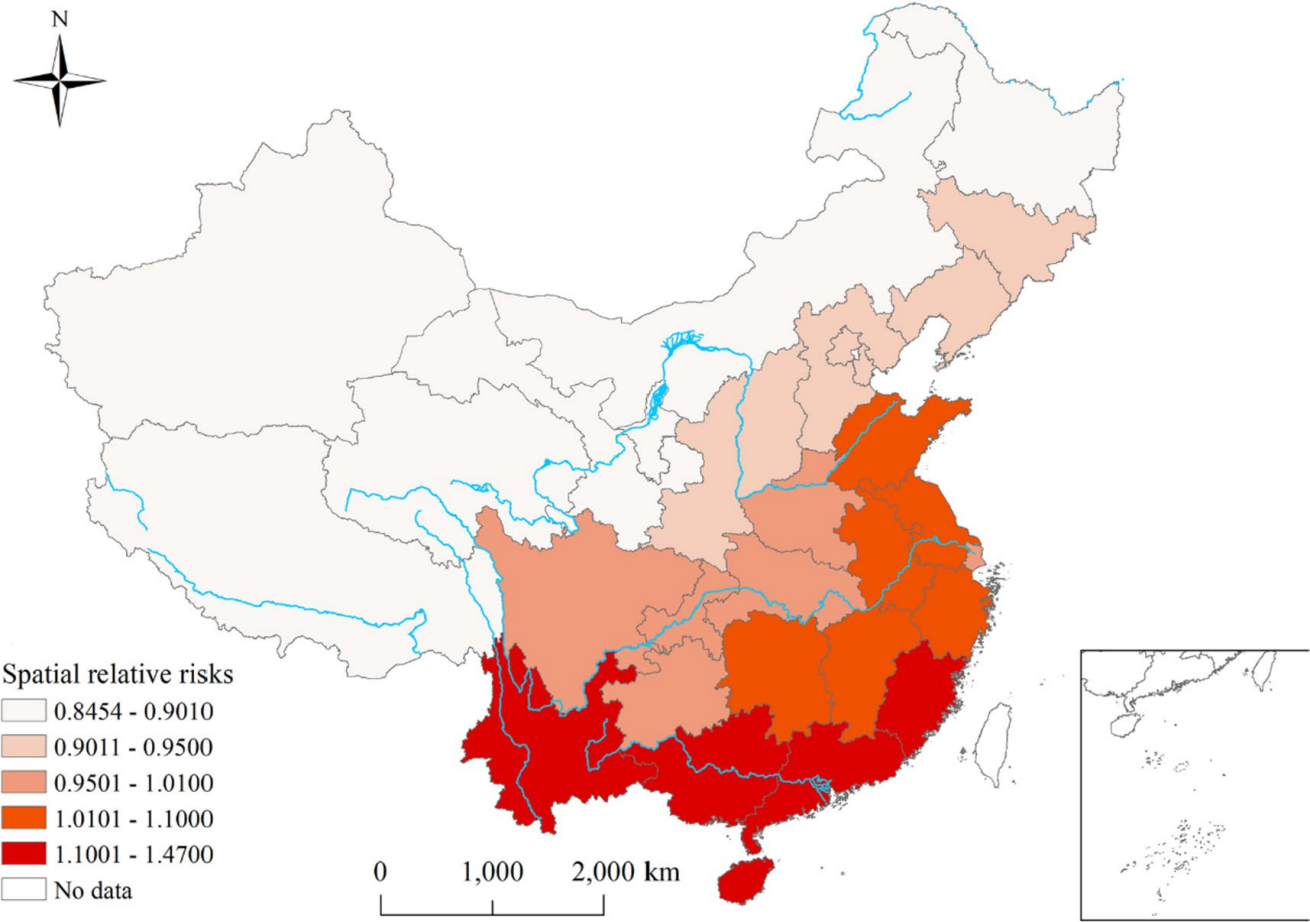
Spatial relative risks of scrub typhus in each province of China. The $\mathrm{exp}({s}_{i})$ is the spatial risk of this disease, which is influenced by some related factors in the study period, such as economic conditions, local prevention and control policies, and medical resources

**Fig. 6:**
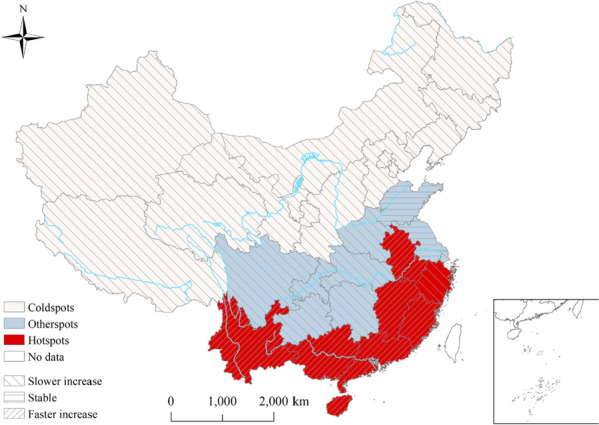
Distribution of hot and cold spots of scrub typhus in China

Geographically, the spatial RRs of scrub typhus calculated from BSTHM indicated clear spatial heterogeneity (Fig. 5). The provinces with higher spatial RRs mainly appeared in southeast China showed higher spatial RRs while the northern provinces in China had lower spatial RRs (Fig. 5). These results suggest that the southern regions involved in higher risks. Based on the posterior probability $P(exp({s}_{i})>1|data)$, the 31 provinces were classified into three categories: hot spots, cold spots, and other spots. Among the 31 provinces, 14/31 (45.16%) were identified as cold spots, while 8/31 (25.81%) were identified hot spots. The remaining 9/31 (29.03%) provinces were classified as neither cold spots nor hot spots (Fig. 6).

Among the eight hot spots, the upward trends of Anhui, Jiangxi, Yunnan, Fujian, Guangdong, Guangxi, and Hainan were faster than the overall trend. As a result, the risk in these regions might be higher than the overall risk and could continue to face high risk in the future. Therefore, it is crucial for relevant prevention and control departments to prioritize these provinces. On the other hand, Zhejiang showed a slower increasing trend compared to the overall trend, suggesting that the risk in Zhejiang will be lower than the overall risk in the future and it may even become a coldspot (Fig. 6).

Among the fourteen cold spots, all provinces showed a lower increase compared to the overall trend. This suggests that the risk in these provinces is expected to be lower than the overall risk, and they are likely to maintain a low-risk state in the future (Fig. 6).

Among the nine remaining provinces without hot spots or cold spots, Henan, Shanghai, Hunan, Guizhou, Chongqing, Sichuan, and Hubei exhibited a slower upward trend, suggesting that these provinces may potentially become cold spots in the future. On the other hand, the other three provinces followed the overall trend (Fig. 6).

### Risk factor analysis

The risk of scrub typhus showed noticeable seasonal variations and spatial heterogeneity, as depicted in Figs. 4 and 6. This suggests that both environmental and socioeconomic factors significantly contribute to the spatiotemporal heterogeneity of scrub typhus. In northern China, the most influential factors were found to be average temperature and illiteracy rate, as indicated in Tables 4 and 6. Conversely, in southern China, the factors with the greatest determinant powers were NDVI and altitude (Tables 5 and 6).

**Table 4:** The quantified posterior mean values with 95%CRI and the relative risks (RRs) for all environmental factors in the northern provinces of China

Environmental factors	Posterior mean(95%CRI) (100%)	RR (95%CRI)
Monthly average temperature, per IQR (℃)	-10.720(-13.780, -7.470)	0.8985(0.8713,0.9280)
Monthly relative humidity, per IQR (%)	-3.042(-4.507, -1.613)	0.9701(0.9559,0.9840)
Monthly precipitation, per IQR (mm)	1.289(0.311, 2.263)	1.0130(1.0030,1.0230)
Monthly total sun hours, per IQR (h)	-3.024(-4.670, -1.442)	0.9702(0.9544,0.9857)
Monthly normalized vegetation index, per IQR (%)	-7.580(-10.960, -4.424)	0.9271(0.8962, 0.9567)
Monthly wind speed, per IQR (m/s)	-3.515(-5.224, -1.879)	0.9655(0.9491, 0.9814)

**Table 5:** The quantified posterior mean values with 95%CRI and the relative risks (RRs) for all environmental factors in the southern provinces of China

Environmental factors	Posterior mean(95%CRI) (100%)	RR (95%CRI)
Monthly average temperature, per IQR (℃)	15.800(9.171,22.190)	1.1720(1.0960,1.2480)
Monthly relative humidity, per IQR (%)	5.342(2.535, 8.047)	1.0550(1.0260,1.0840)
Monthly precipitation, per IQR (mm)	-4.908(-10.740,1.237)	0.9525(0.8982,1.0120)
Monthly total sun hours, per IQR (h)	3.897(-0.117,8.975)	1.0400(0.9988,1.0940)
Monthly normalized vegetation index, per IQR (%)	19.180(6.976, 30.840)	1.2140(1.0720, 1.3610)
Monthly wind speed, per IQR (m/s)	-2.375(-4.850, 0.647)	0.9766(0.9527, 1.0060)

**Table 6:** The *q* values (*q*_1_, *q*_2_) calculated for the association between scrub typhus and altitude and socioeconomic factors in northern and southern China, respectively

Socioeconomic factors	q_1_(northern)	q_2_(southern)
Urbanization rate (%)	0.417^a^	0.540^a^
Percentage of population over 65(%)	0.505^a^	0.594^a^
Per capita GDP (10^4^CNY)	0.740^a^	0.542^a^
Population density(person/km^2^)	0.748^a^	0.297^a^
Provincial-level altitude(m)	0.768^a^	0.882^a^
Illiteracy rate (100%)	0.844^a^	0.504^a^
Number of medical beds (bed)	0.279^a^	0.852^a^
Percentage of population aged 0–14(%)	0.280^a^	0.569^a^
Number of health technicians (per 10^3^)	0.167^a^	0.707^a^

^a^Statistical significance level: 0.01

In the northern region of China, temperature was identified as the most influential factor, having a strong correlation with the risk of scrub typhus. A 1-IQR increase in temperature was found to be associated with a significant 10.720% decrease in the risk of scrub typhus, as indicated by a corresponding RR value of 0.8985 (0.8713–0.9280) (Table 4).

Precipitation was found to have a positive correlation with scrub typhus risk. Specifically, for every interquartile range (IQR) increase in precipitation, there was a corresponding 1.289% increase in scrub typhus risk. This relationship was further supported by a relative risk (RR) value of 1.013(1.003–1.023) (Table 4).

The effect of other potential meteorological factors could not be ignored. For example, per IQR increase in wind speed was associated with a decrease of 3.515% in the risk of scrub typhus (RR: 0.9655; 95% CRI: 0.9491–0.9814) (Table 4).

Per IQR increase in RH was associated with a 3.042% risk reduction (RR: 0.9701; 95% CRI: 0.9559–0.9840). A 1-IQR increase in total solar hours was related to a 3.024% decrease in scrub typhus risk, with a corresponding RR of 0.9702(95% CRI: 0.9544–0.9857) (Table 4).

NDVI also had a nonnegligible effect. Per IQR increase in NDVI was associated with a reduction of 7.580% in scrub typhus risk (RR: 0.9271; 95% CRI: 0.8962–0.9567) (Table 4).

The results of q statistics in Geodetector indicate that geographical and socio-economic factors also played significant roles in the transmission of scrub typhus. In northern China, the illiteracy rate was had the highest determinant power, with a *q* value of 0.844. Altitude, the percentage of the population over 65, and per capita GDP have determinant powers of 0.768, 0.505, and 0.740, respectively. The determinant powers of the percentage of the population aged 0–14, population density, and the urbanization rate were 0.280, 0.748, and 0.417, respectively. Additionally, the determinant powers of the number of medical beds, and the number of health technicians are 0.279, and 0.167, respectively (Table 6).

In the south, NDVI had the most important effect on scrub typhus and was positively associated with the risk of scrub typhus. Per IQR increase in NDVI was related to a 19.180% increase in scrub typhus, with a corresponding RR value of 1.214 (95%CRI:1.072, 1.361) (Table 5).

Per IQR increase in temperature was associated with an increase of 15.8% in the risk of scrub typhus (RR: 1.172; 95% CRI: 1.096–1.248) (Table 5).

In addition to the average temperature and NDVI, relative humidity (RH) also had significant impact on the occurrence of this disease. A per interquartile range (IQR) increase in RH was associated with a 5.342% increase in scrub typhus risk, with a corresponding RR value of 1.055 (95% CRI: 1.026–1.084). In addition, the estimated coefficient of precipitation, sun hours, and wind speed showed no the prevalence of scrub typhus in southern China (Table 5).

The Geodetector analysis revealed that attitude had the highest determinant power, with a *q* value of 0.882. The determinant powers of the number of medical beds, per capita GDP, and the percentage of population aged 0–14 were 0.852, 0.542, and 0.569, respectively. Additionally, the determinant powers of the percentage of population over 65, urbanization rate, and number of health technicians were 0.594, 0.540, and 0.707, respectively. Lastly, the determinant powers of population density and the illiteracy rate were 0.297 and 0.504, respectively (Table 6).

## Discussion

In this study, we utilized the BSTHM and GeoDetector model to comprehensively assess the spatiotemporal heterogeneity and risk factors associated with scrub typhus from 2006 to 2018. The results revealed a clear spatiotemporal heterogeneity in the scrub typhus risk, with hot spots primarily concentrated in southeast China, while cold spots were predominantly found in northern China. Moreover, temperature and illiteracy rate emerged as the dominant factor influencing scrub typhus risk in northern China, whereas NDVI and altitude were the primary influencing factors in southern China.

The spatial distribution of the risk of scrub typhus risk in China was uneven, with high-risk areas primarily located in the southeast (Anhui, Zhejiang, Jiangxi, Fujian, Guangxi, Yunnan, Hainan, and Guangdong). The vast territory of China has resulted in significant regional variations, and disparities in environmental and socioeconomic factors between the north and south have contributed to varying levels of scrub typhus risk.

On one hand, differences in environmental factors, such as the number and species of chigger mites and rodents, may contribute to the variations in vectors and hosts between the two regions. In the 1950s, Guangdong and Fujian in the south were identified as natural foci of this disease [10, 11], indicating a favorable environmentfor the transmission of scrub typhus. Derne et al.’s study also further highlighted that the complex and diverse terrain, along with high biodiversity in the southern landscape, provide suitable habitats for chigger mites and rodent hosts [35]. Our study revealed that the Normalized Difference Vegetation Index (NDVI) played a crucial role in determining the risk of scrub typhus, suggesting a close relationship between the disease and vegetation. Additionally, the results indicated a significant correlation between altitude and scrub typhus, as the difference in altitude influenced the NDVI and subsequently affected the occurrence of scrub typhus.

However, it is important to note that socio-economic factors also played a significant role. The expansion of urban parks, greening, and river, which were part of the general trend of urbanization and eco-friendly cities, inadvertently increased the risk of residents contracting scrub typhus [36]. In fact, the expansion of urban parks in South Korea even created potential breeding grounds for the disease in urban areas [37].

The susceptibility to infection is higher among the elderly compared to the young. The aging population has result in an increased proportion of individuals over 65 years old, thereby elevating the risk of scrub typhus. Additionally, the growth of trade and tourism, coupled with the continuous development of the economy, has facilitated the transmission of vector-borne pathogens. A study conducted on the Penghu Islands showed a correlation between higher school enrolment and a reduction in rate of child disease infection [38]. The study’s finding also highlighted the significance of the percentage of children under 14 years old and the illiteracy rate as important factors influencing the risk of scrub typhus. Notably, children and students ranked among the top 4 occupational groups affected by scrub typhus, emphasizing the need to prioritize their education and implement preventive measures [11].

Scrub typhus also presented obvious temporal heterogeneity. The high-risk seasons were summer (June to August) and autumn (September to November), especially autumn, which was consistent with the results observed in some previous studies [39, 40]. It may be related to the dominant vector and animal host of scrub typhus. For example, the reproductive peak of *Rattus losea* was from March to October, with the highest in October, and the lowest in February, which was similar to the risk change of scrub typhus. *Chigger mites* were both hosts and vectors, mostly distributed in provinces and regions from the southeast coast to the southwest border of China. *L. deliense* was widely distributed in the south of 30° north latitude in China and was the main vector of summer-type scrub typhus. As the second largest vector of scrub typhus in China and the main vector of autumn and winter-type scrub typhus, *L. scutellare* was widely distributed throughout the country [25]. In China, the hosts of *Orientia tsutsugamushi* were mainly *Rattus losea*, *Rattus tanezumi*, *Rattus norvegicus*, *Nivirenter confucianus*, and *Apodemus agrarius* [41], of which *Rattus losea* and *Rattus tanezumi* were mainly distributed in the south. This seasonal change and spatial heterogeneity will help the public health department formulate reasonable prevention and control policies and allocate resources according to the peak incidence and the months with more reported cases reasonably.

Meteorological factors were considered important environmental factors, which had a complex impact on the epidemic of scrub typhus [20, 42]. The climate difference between the north and the south also led to the temporal and spatial heterogeneity of scrub typhus. In the north, the average temperature, relative humidity, wind speed, and sunshine hours were negatively correlated with the risk of scrub typhus, while precipitation was positively correlated with the risk of scrub typhus. On the contrary, in the south, the average temperature and relative humidity were positively correlated with scrub typhus, indicating that the meteorological range was a decisive factor.

The complex interactions between environment, vector, and human could explain these results. Firstly, the difference of dominant mite species between the north and the south was one of the reasons for this opposite result. *L.deliense* was the dominant mite species in the south, which can complete the whole life cycle process in the temperature range of 13 ± 1 – 35 ± 1° C [43]. In addition, some experimental studies showed that the optimum temperature for the development and reproduction of this species seemed to be 18 – 28° C and 23 ± 1° C seems to be the optimum temperature for larval hatching [43]. At a constant temperature of 25 ± 1° C, the survival time of *L. deliense* increased with the increase of humidity. However, the warm and humid environment was not conducive to the survival, development and reproduction of *L. scutellare*, the dominant mite species in the north [44, 45]. *L. scutellare had strong resistance to cold environments, and can survive for 2 months at 1–2° C, or even one month at—20° C* [46].

Secondly, climate range was an important factor affecting the main carrier and host of scrub typhus [47]. Lu et al.’ research showed that before 29.6 ℃, with the increase in temperature, the oviposition rate of *chiggeridae* and the number of rodents increased, and *Chigger mites* became more active [48]. The RR value was the highest at 27℃, and the weekly temperature range was negatively correlated with the risk of disease [49]. When the temperature was too low or too high, Chigger mites were difficult to feed, and the chances of attaching to humans will be reduced [47, 50], while mild climate may trigger eggs to develop into larvae, thus increasing exposure risk [51]. Meanwhile, higher humidity was also not conducive to the life cycle of Chigger mites. For example, Yao et al. study [11] found that southern areas with relative humidity higher than 63% had a lower risk than other areas, while the humidity in the northern region was between 62%-65%, which easily led to the risk of disease. Therefore, a detailed monitoring of environmental humidity possibly helped to provide a fine map of the future risks of scrub typhus.

Finally, climate conditions affect the frequency of people’s outdoor activities, thus changing the contact with the vectors [11]. Warming may enhance agricultural and recreational activities, leading to high-risk exposure [11], while the temperature is higher, people will reduce their willingness to farm or go out (such as to parks), thus reducing the risk of tsutsugamushi infection.

In northern China, wind speed had a negative correlation with the impact of scrub typhus, while in southern China, the correlation was not significant. Few studies have discussed the relationship between wind speed and scrub typhus. A study in Fujian by Li et al. showed that wind speed was negatively correlated with scrub typhus, which was not consistent with the impact of wind speed in the south on scrub typhus in this study [52]. The reasons for these phenomena may be: firstly, the regional characteristics of different research areas may lead to changes in climate conditions, which had different impacts on the epidemiology of scrub typhus. Secondly, climatic conditions were only some factors related to scrub typhus; Human factors were also indispensable in the transmission of infectious diseases. A South Korean study showed that wind speed may be related to the spawning conditions of tsutsugamushi [53]. Gale weather was usually accompanied by rain, which may affect the reduction of the number of people engaged in agriculture or other outdoor activities in contact with vegetation. However, the impact of wind speed on tsutsugamushi ecology and life cycle should be further studied in the future.

The study found that precipitation was positively correlated with the risk of scrub typhus in the north, which was consistent with the research results of Yao et al. [11]. Rainfall in the north may increase the number of tsutsugamushi, thus increasing the risk of exposure. However, the humidity in the south was high, and the precipitation increased the humidity again, which was not conducive to the survival of the larvae.

Some previous studies showed that sunshine was also a dominant factors determining the activity time of Chigger mite larvae [54], which may partly explain the lower incidence in northern China than in the south. The results showed that the number of sunshine hours was positively correlated with scrub typhus within a certain range, but showed an inhibitory effect when it exceeded this range. For example, in Yao et al. [11] BRT model, sunshine hours of less than 170 were positively correlated with the risk of scrub typhus, while sunshine hours of more than 170 were negatively correlated with the disease. The average sunshine duration in northern China was 197.91, which was much higher than the average sunshine duration in southern China of 146.46. The research of Yang et al. also showed that the sunshine duration was a negative factor in Shandong in the north [55]. This study has some limitations. Firstly, only the cases seeking medical care in the hospital were reported to the local disease prevention and control center and used for analysis, so the current analysis omits the clinical cases without doctors or the subclinical infections. Secondly, as a multifactorial disease, factors other than those considered in this study, such as tourist trade, human behavior patterns, public health policy, mite epidemic data, and pathogen genotypes, all may be related to epidemiological heterogeneity. Finally, new factors such as the prevalence of emerging infectious diseases will also affect the spread of scrub typhus. However, the current research results can comprehensively compare the epidemic characteristics and influencing factors of scrub typhus in the northern and southern regions. In the later control and prevention of scrub typhus, the differences in different regions can be considered and resources can be reasonably allocated.

## Conclusions

This study investigated the temporal and spatial heterogeneity of scrub typhus in southern and northern China from 2006 to 2018, focusing on the dominant forces of meteorological, environmental and socio-economic factors. The finding revealed significant seasonality and spatial variations in scrub typhus. The disease primarily occurred during the summer and autumn seasons, with high-risk areas concentrated in southeast China. These differences attributed to the complex interplay between the environmentand human populations. In northern China, temperature and illiteracy rate emerged as the dominant factors, whereas NDVI and altitude played a more influential role in southern China. The study highlights the importance of strengthing the monitoring efforts on the ecological environment, hosts, and vectors of *Orientia tsutsugamushi*, as well as enhancing risk awareness, particularly among vulnerable groups such as children and the elderly. This is crucial for preventing and controllingthe potential increase in scrub typhus risk under these meteorological, environmental and socio-economic conditions. Furthermore, considering the regional differences, it is essential to allocate resources effectively.

### Supplementary Information

**Additional file 1.**

**Additional file 2.**

**Additional file 3.**

**Additional file 4.**

## Data Availability

Data supporting the results of this study may be obtained from the corresponding authors upon reasonable request.
