# Supplementary material for: Spatiotemporal heterogeneity and long-term impact of meteorological, environmental, and socio-economic factors on scrub typhus in China from 2006 to 2018
Source: BMC Public Health. 2024 Feb 21;24:538. doi: 10.1186/s12889-023-17233-y (PMC10880311; doi:10.1186/s12889-023-17233-y)
Supplement: Supplementary file 2 — Additional file 2. [file 12889_2023_17233_MOESM2_ESM.doc]

**Legends:The definition of scrub typhus is based on the unified diagnostic standards formulated by the Chinese Ministry of Health.**

A suspected case was defined as follows: (a) an individual with a clear epidemiological history (travelling to endemic areas or contact with chiggers or rodents within three weeks prior to the onset), clinical manifestations (acute fever, skin rash, eschars or ulcers, and lymphadenopathy), and an agglutination titer ≥ 1:160 in the Weil-Felix test using the OXK strain of Proteus mirabilis.

Laboratory confirmed cases are defined as possible cases with at least one laboratory standard: a fourfold or greater rise of serum IgG antibody titers between acute and convalescent sera detected by indirect immunofluorescence antibody assay (IFA), detection of *O. tsutsugamushi* in clinical samples by polymerase chain reaction (PCR), or isolation of *O. tsutsugamushi* from clinical samples.
