# Supplementary material for: Spatiotemporal heterogeneity and long-term impact of meteorological, environmental, and socio-economic factors on scrub typhus in China from 2006 to 2018
Source: BMC Public Health. 2024 Feb 21;24:538. doi: 10.1186/s12889-023-17233-y (PMC10880311; doi:10.1186/s12889-023-17233-y)
Supplement: Supplementary file 3 — Additional file 3. [file 12889_2023_17233_MOESM3_ESM.doc]

**Legends:****The selection of specific discretization method and interrupt count for data in Geodetector.**

| Region/Variables | Southern | | Northern | |
| --- | --- | --- | --- | --- |
|  | Discretization method | number of intervals | Discretization method | number of intervals |
| Number of health technicians (per 10^3^) | quantile | 4 | quantile | 4 |
| Illiteracy rate (100%) | sd | 4 | sd | 4 |
| Percentage of population aged 0-14(%) | quantile | 4 | quantile | 4 |
| Population density(person/km^2^) | sd | 4 | sd | 4 |
| Percentage of population over 65(%) | natural | 4 | natural | 4 |
| Provincial-level altitude(m) | geometric | 3 | geometric | 3 |
| GDPR | sd | 4 | sd | 4 |
| Urbanization rate (%) | equal | 4 | equal | 4 |
| Number of medical beds (bed) | equal | 3 | equal | 3 |
