# Supplementary material for: Spatiotemporal heterogeneity and long-term impact of meteorological, environmental, and socio-economic factors on scrub typhus in China from 2006 to 2018
Source: BMC Public Health. 2024 Feb 21;24:538. doi: 10.1186/s12889-023-17233-y (PMC10880311; doi:10.1186/s12889-023-17233-y)

Supplementary figures of Fig. 3

Title: Monthly incidence and annual incidence of scrub typhus in 31 provinces of China.


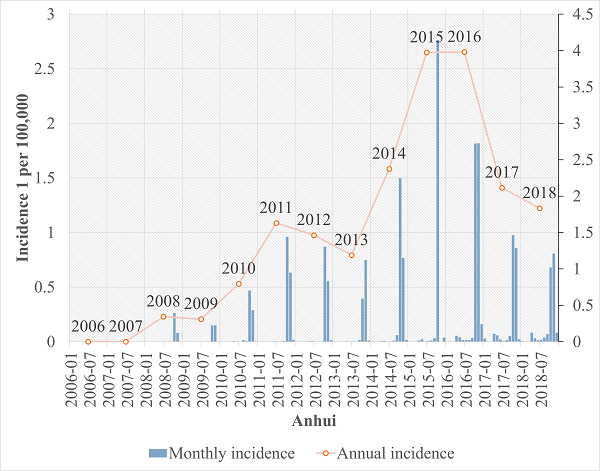


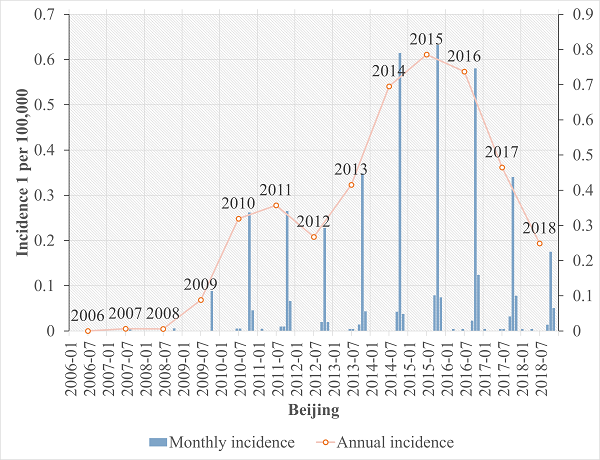


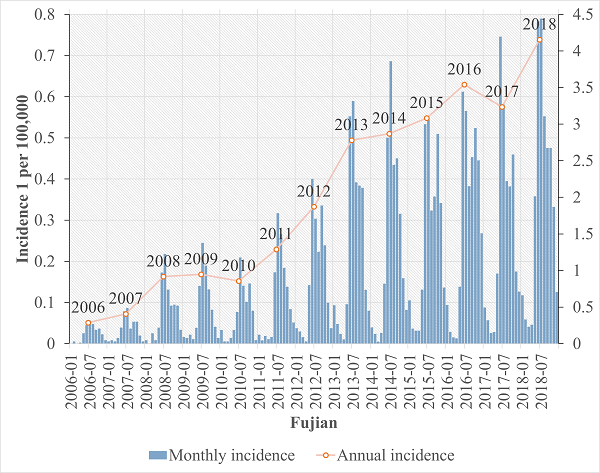


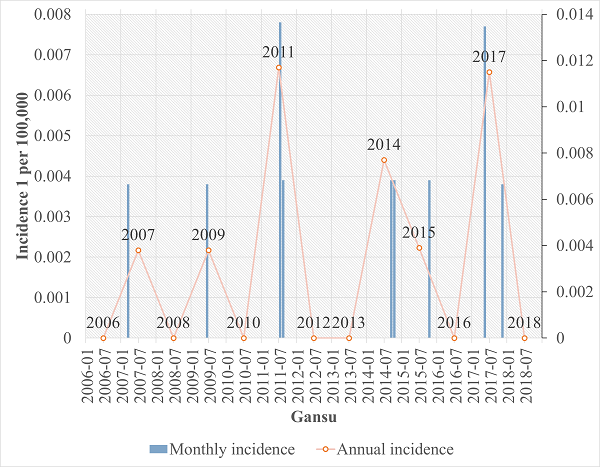


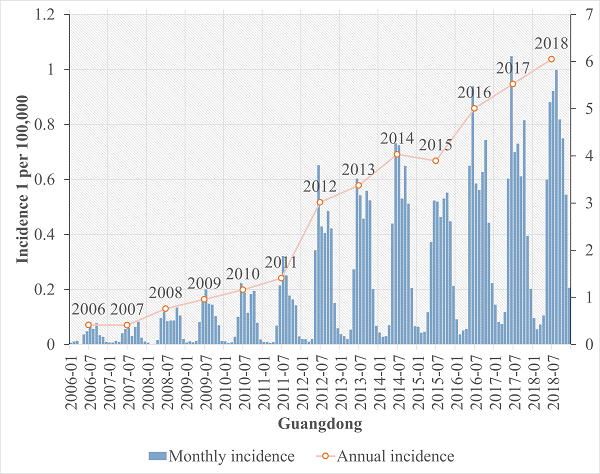

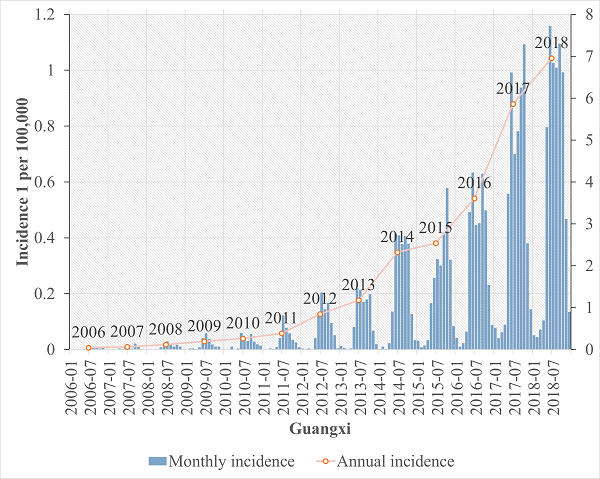


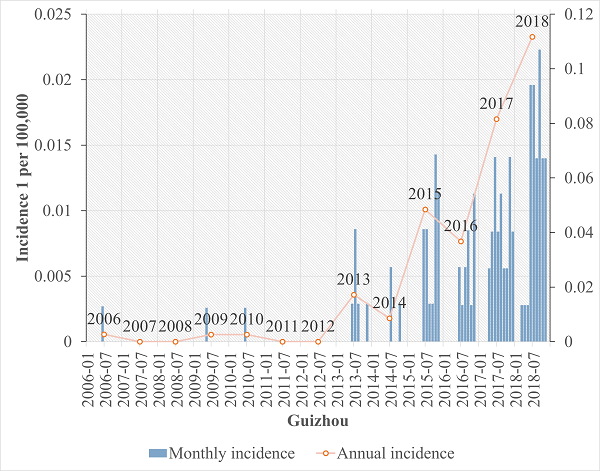


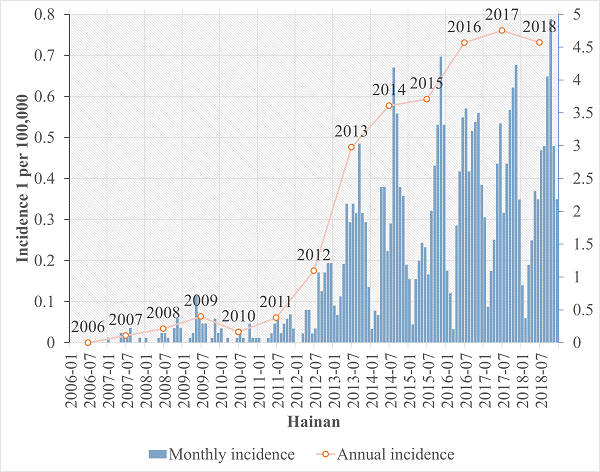


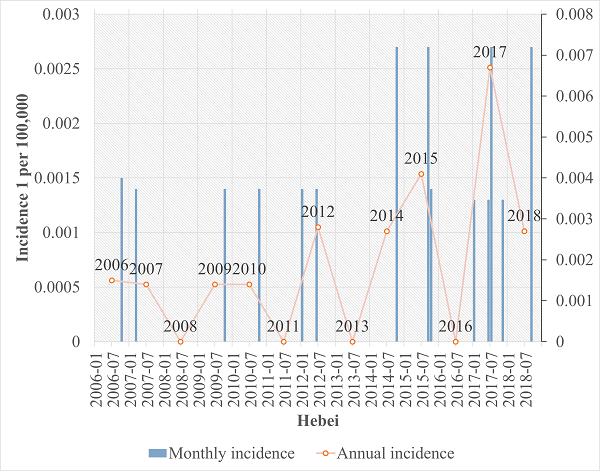


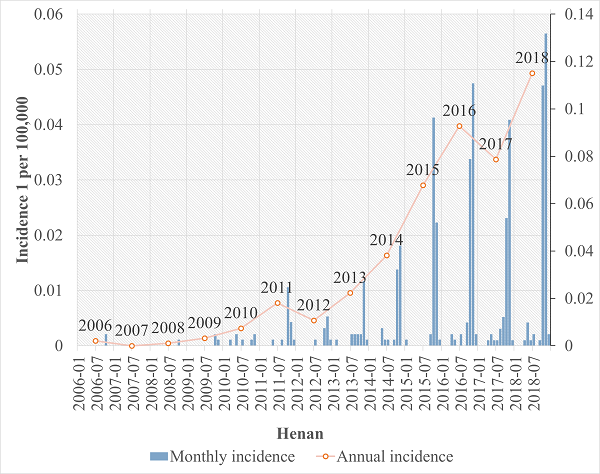


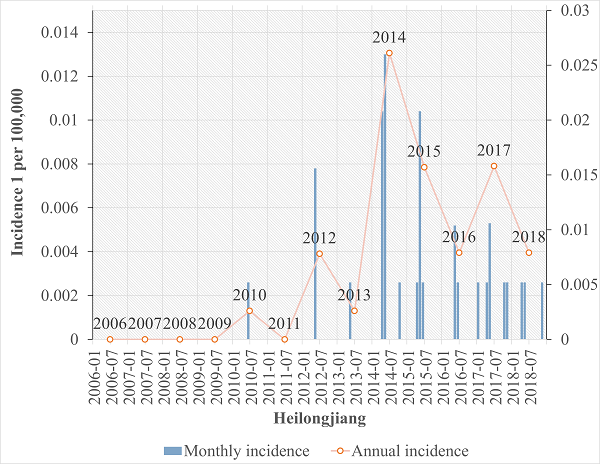


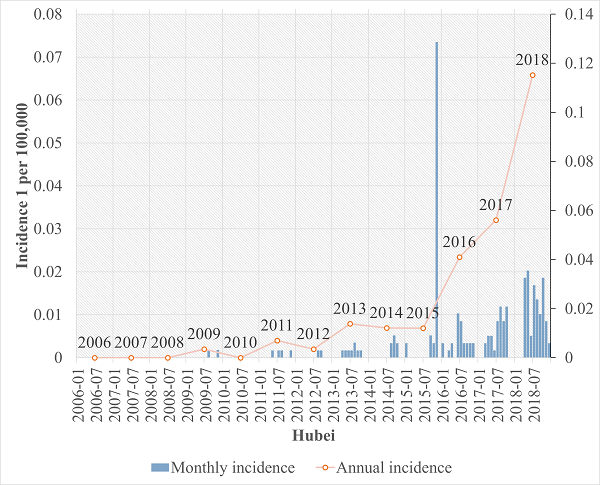


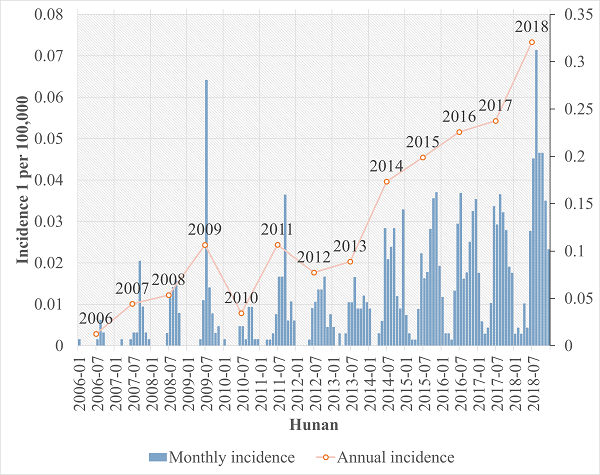


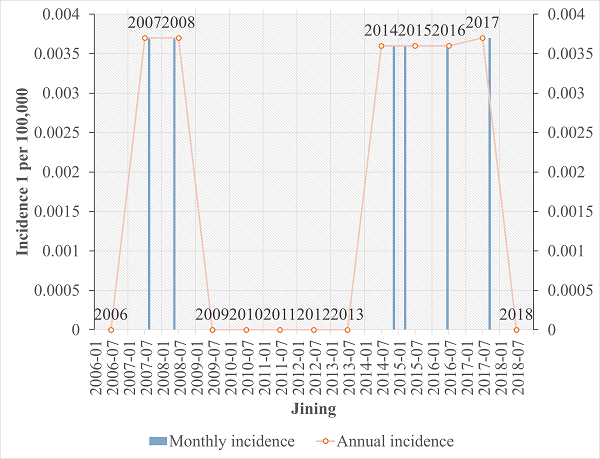


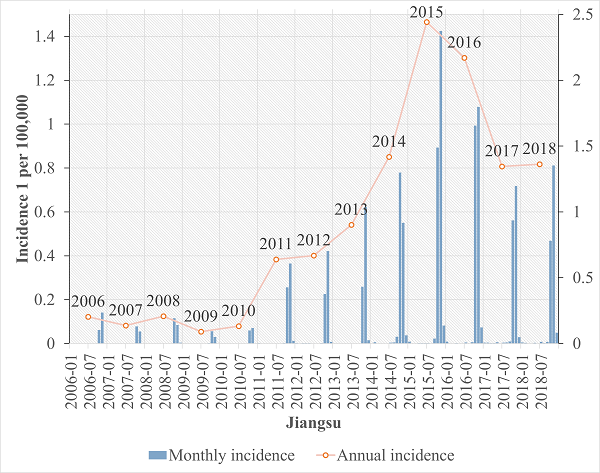


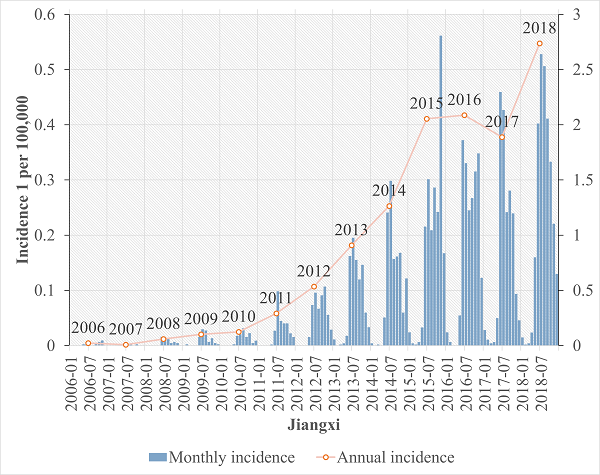


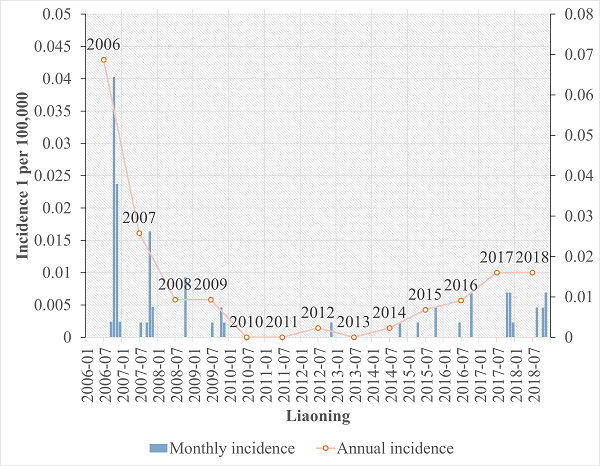


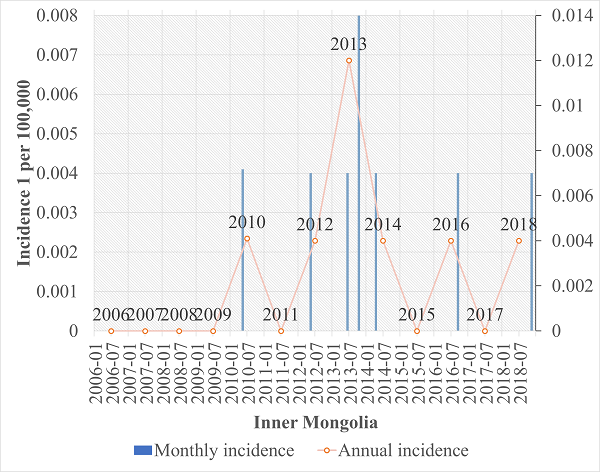


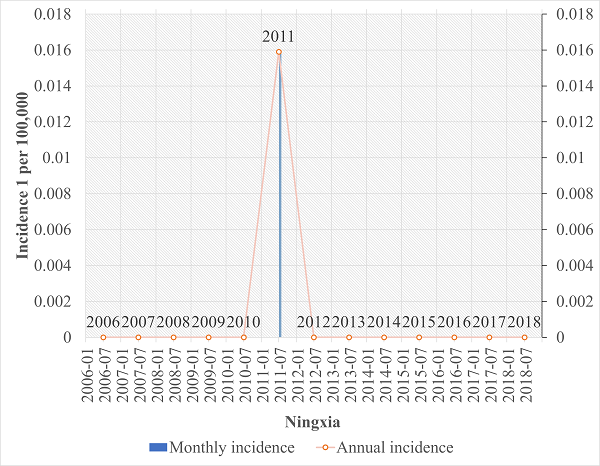


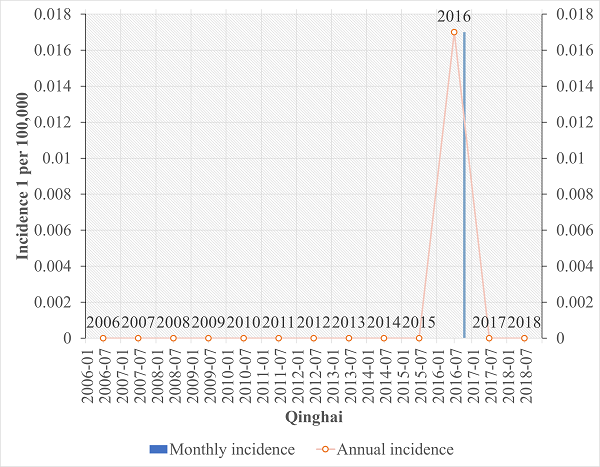


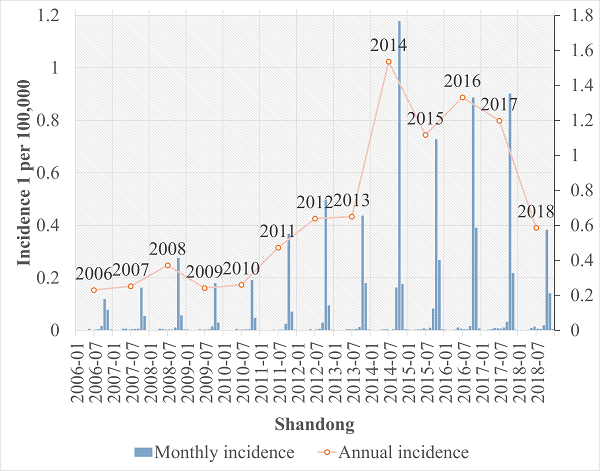


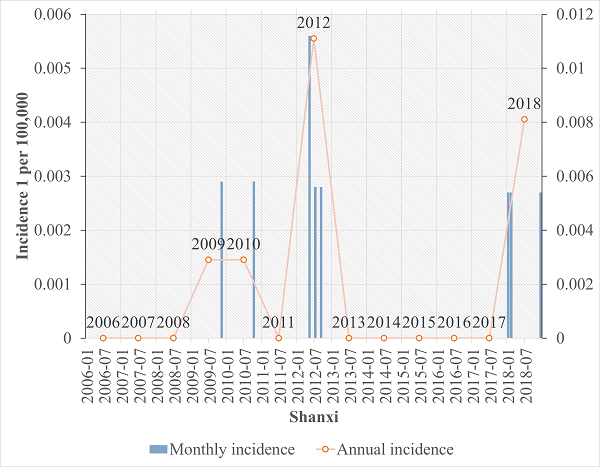


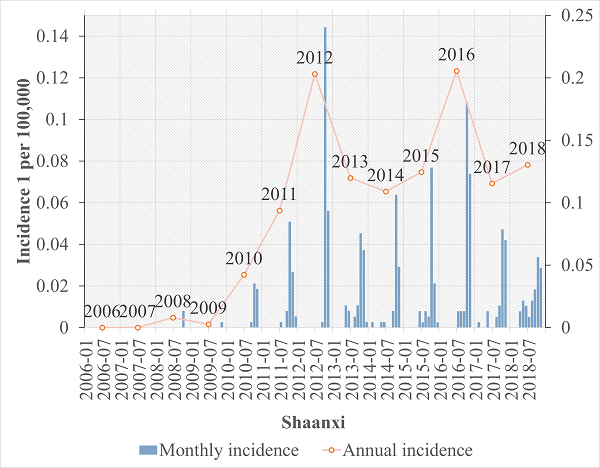


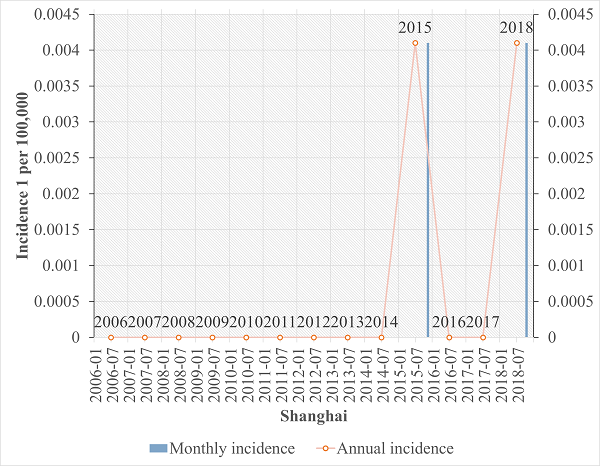


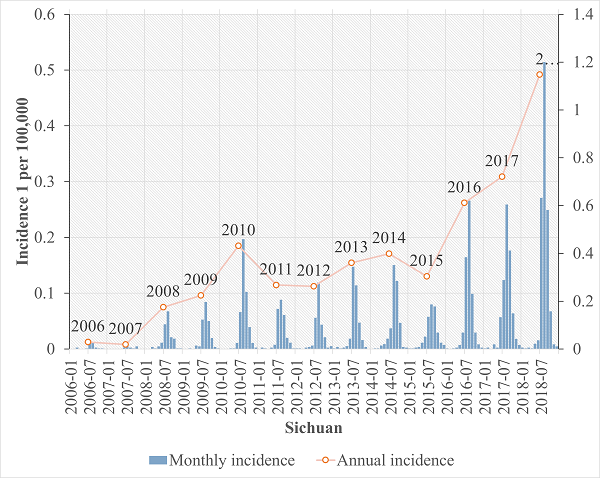


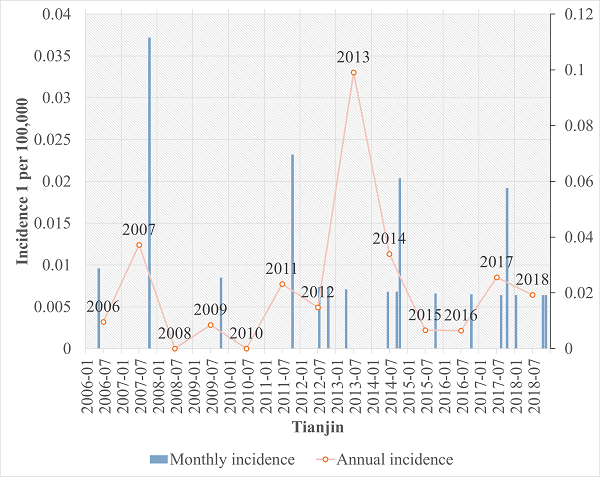


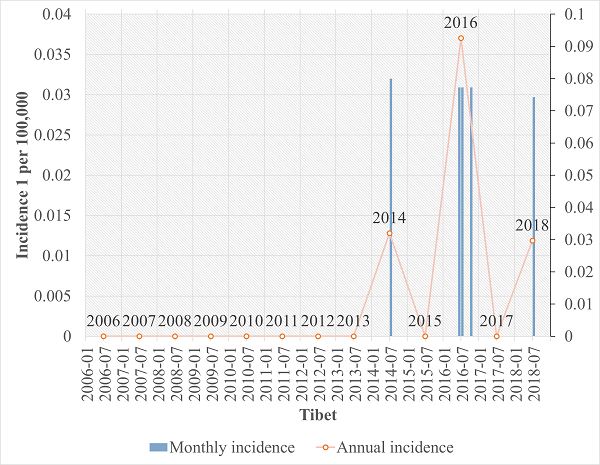


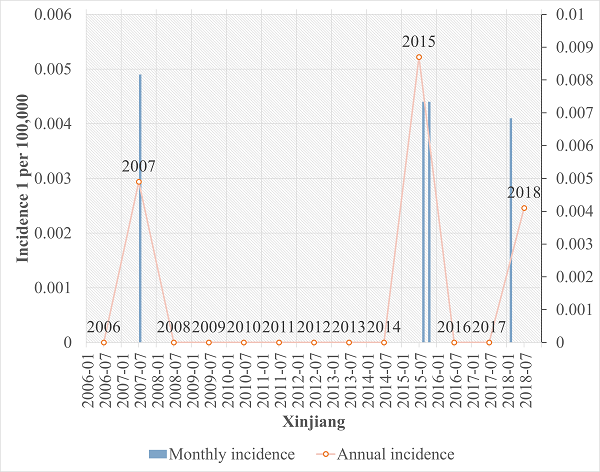


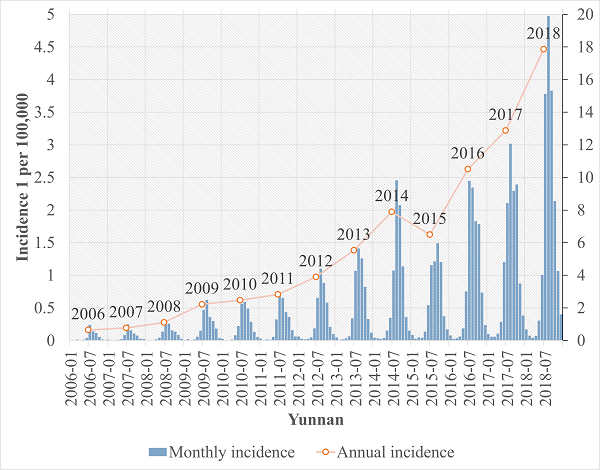


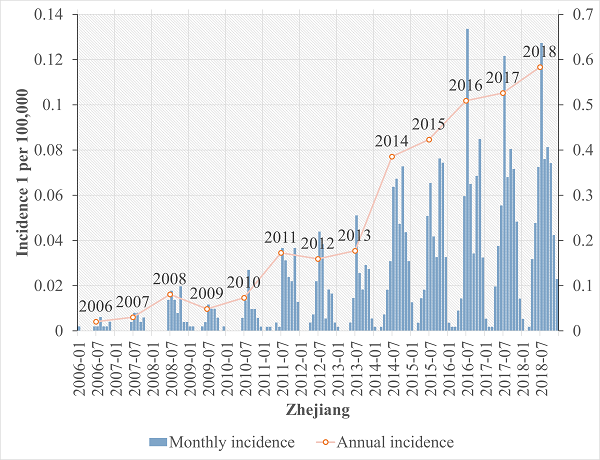


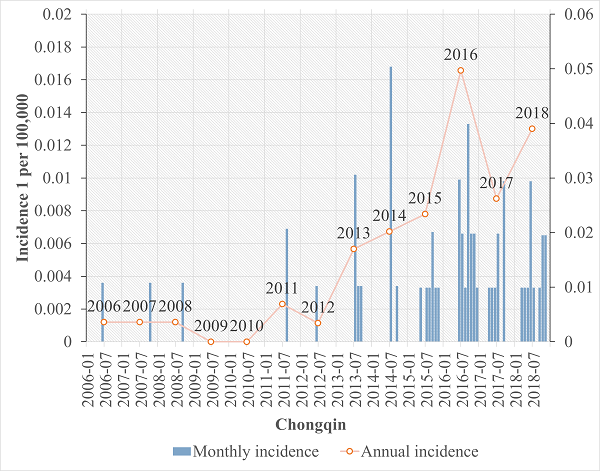

Supplement: Supplementary file 4 — Additional file 4. [file 12889_2023_17233_MOESM4_ESM.doc]
